# *Escherichia coli* hijack Caspr1 receptor to invade cerebral vascular and neuronal hosts

**DOI:** 10.15698/mic2018.09.647

**Published:** 2018-08-07

**Authors:** Wei-Dong Zhao, Dong-Xin Liu, Yu-Hua Chen

**Affiliations:** 1 Department of Developmental Cell Biology, Key Laboratory of Cell Biology, Ministry of Public Health, and Key Laboratory of Medical Cell Biology, Ministry of Education, China Medical University, 77 Puhe Road, Shenbei New District, Shenyang 110122, China.

**Keywords:** Caspr1, blood-brain barrier, IbeA, Escherichia coli, bacterial meningitis

## Abstract

*Escherichia coli* (*E. coli*) penetration of the blood-brain barrier (BBB) is the key step essential for the development of meningitis. In a recent paper (Nat Commun 9:2296), we identify Caspr1 as a host receptor for *E. coli* virulence factor IbeA to pave the way the penetration of bacteria through the BBB. Bacterial IbeA interacts with endothelial Caspr1 to trigger intracellular focal adhesion kinase activation, leading to *E. coli* internalization into the brain endothelial cells. Importantly, endothelial knockout of Caspr1 in mice significantly reduced *E. coli* crossing through the BBB. Based on the results that extracellular aa 203-355 of Caspr1 bind with IbeA, we tested the blocking effect of recombinant Caspr1(203-355) peptides in neonatal rat model of meningitis. The results showed that Caspr1(203-355) peptides effectively attenuated *E. coli *penetration into the brain during meningitis, indicating that Caspr1(203-355) peptides could be used to neutralize the virulent IbeA to prevent meningitis. We further found that *E. coli* can directly invade into hippocampal neurons causing apoptosis which required the interaction between bacterial IbeA and neuronal Caspr1. These findings demonstrate that *E. coli *hijack Caspr1 as a host receptor for penetration of BBB and invasion of hippocampal neurons, resulting in progression of meningitis.

## INTRODUCTION

*E. coli* with K1 capsule is the primary bacteria that cause neonatal bacterial meningitis. In the past decades, microbiologists identified several virulence factors in *E. coli* K1 strain that were involved in bacterial meningitis. Further characterization of these bacterial virulence factor in the context of pathogen-host interaction is necessary to elucidate the molecular mechanism of *E. coli* meningitis (ECM), which is helpful to design novel strategies for therapeutic interventions.

**Figure 1:**
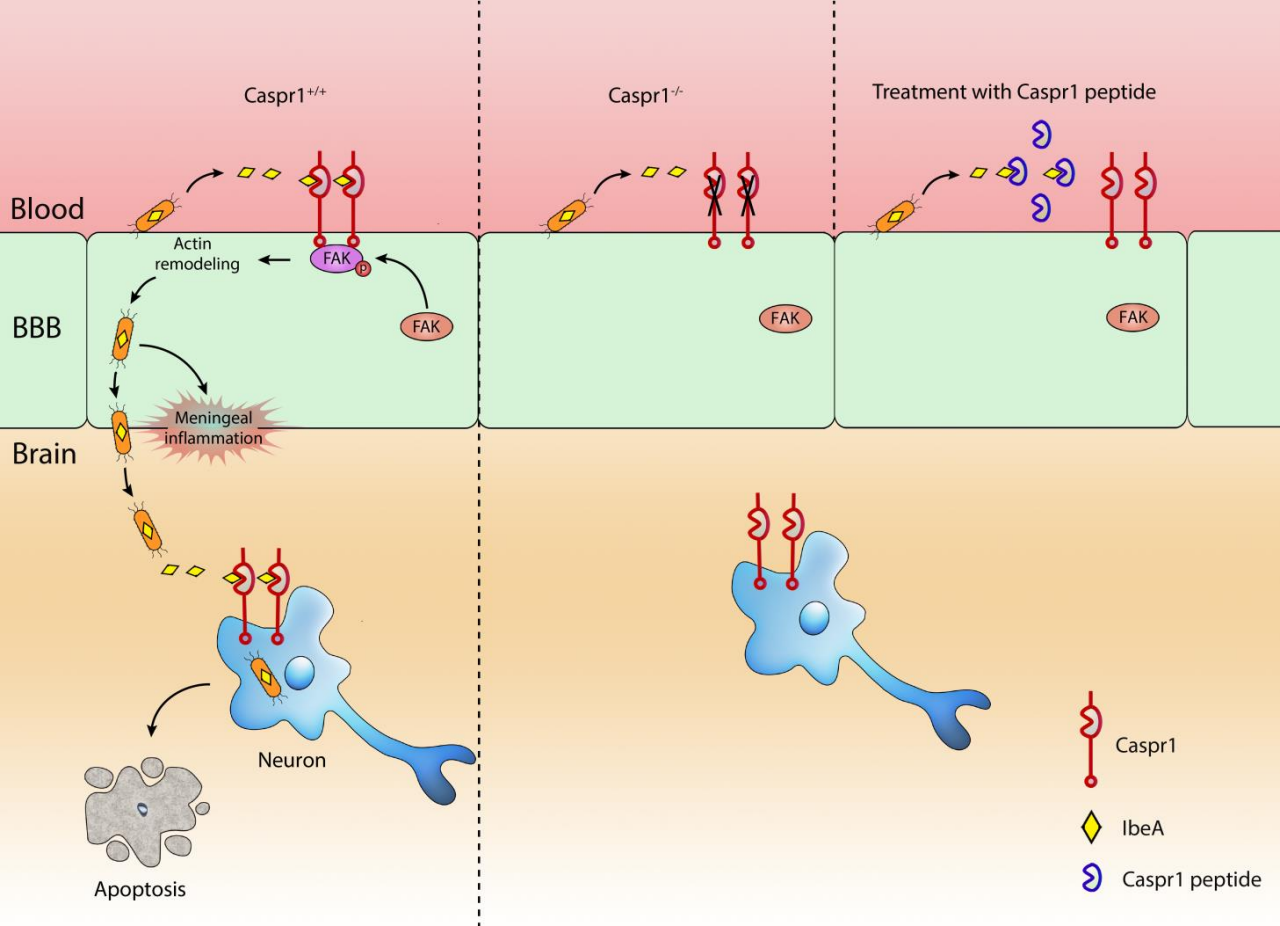
FIGURE 1: Bacterial IbeA interacts with host Caspr1 to facilitate development of meningitis. The virulence factor IbeA is secreted from *E. coli *upon contact with brain endothelium during bacteremia. The soluble IbeA binds with luminal Caspr1 to activate FAK signaling. Then the *E. coli* are internalized into brain endothelial cells and penetrate through the BBB leading to meningeal inflammation. The penetrated *E. coli* outside the microvessels in the brain parenchyma can invade neurons leading to apoptosis, dependent on IbeA-Caspr1 interaction. When Caspr1 was deleted by genetic knockout or blocked with Caspr1 peptide specifically bound with IbeA, the penetration of *E. coli* through the BBB was effectively prevented due to attenuated IbeA-Caspr1 interactions.

IbeA was first identified as a critical determinant of *E. coli* K1 to facilitate bacterial penetration through the blood-brain barrier (BBB). However, the molecular mechanism by which IbeA exerts its effect long remained unknown. With electron microscopy and western blot analysis, we found that IbeA protein was secreted from *E. coli *bodies into the extracellular environment upon contact with host cells, i.e., brain microvascular endothelial cells (BMECs) which is the primary component of BBB. These prompted us to hypothesize that the secreted IbeA may interact with membrane proteins expressed on host cell surface. Then, we used a yeast two-hybrid system to screen the interacting proteins of IbeA in human brain microvascular endothelial cells (HBMECs). Fortunately, we identified Caspr1 (contactin associated protein 1), anchored at the plasma membrane of BMECs, as a novel binding partner of bacterial IbeA. We found that Caspr1 is specifically expressed in brain endothelium, and is localized at the luminal side, as revealed by immunoelectron microscopy. Then we asked whether Caspr1 was involved in bacterial penetration of BBB, which is the crucial step for meningitis development. To obtain genetic evidence, transgenic mice with conditional knockout of Caspr1 in vascular endothelium (Caspr1 eKO) were generated, and we found that Caspr1 eKO significantly reduced the occurrence rate of ECM. To our knowledge, this is the first in vivo evidence regarding a host receptor for ECM. Consistently, a set of in vitro experiments with HBMECs supported that Caspr1 is necessary for *E. coli* invasion into its mammalian host cells, namely brain endothelial cells. These data established the pivotal role of endothelial Caspr1 in bacterial penetration of BBB during ECM (Figure 1).

Naturally, the prominent function of a receptor is to transduce signals from the extracellular environment into the intracellular compartment. To screen the intracellular signaling molecules associated with Caspr1, a pull-down experiment was performed using Caspr1 intracellular domain as a bait, followed by mass spectrometry. In this approach, FAK (focal adhesion kinase) was identified as a candidate interacting molecule. Further results indicated that FAK is recruited to Caspr1 and phosphorylated at site Y397 in response to *E. coli* infection. The downstream signaling molecule of FAK was identified as the small GTPase, Rac1. Based on these findings, we were able to name Caspr1 as the host “receptor” of *E. coli* IbeA essential for ECM. One remaining question is, whether Caspr1 interacts with FAK directly, or whether there is an adaptor protein required for their association. It will be interesting to test these ideas in future studies.

We mapped the interacting domains in Caspr1 and IbeA, respectively. Results from GST pull-down assays showed that the extracellular aa 203-355 domain of Caspr1 interacts with aa 229-342 of IbeA. Then we considered whether this short 203-355 domain of Caspr1 could be used to neutralize the secreted virulent IbeA during meningitis. Thus, recombinant Caspr1(203-355) peptide was purified and tested in neonatal rats with ECM. Interestingly, pretreatment with Caspr1(203-355) peptide reduced the occurrence rate of meningitis in neonatal rats. These results presented Caspr1(203-355) peptide as a novel alternative strategy to prevent the development of ECM. However, we noticed that the rate of meningitis was reduced to only ~50% of the control (56% to 24%), suggesting that Caspr1 may be not the only receptor required for meningitis-causing* E. coli*. Except for Caspr1, there might be other unrecognized receptor-ligand interactions involved in ECM. Another possible reason is the compensatory roles of other members of Caspr family (Caspr-2 to Caspr-5), which is worth studying in the future.

Why do *E. coli* favor Caspr1 during meningitis? We think there are 3 major reasons: 1) Caspr1 is exclusively present in the brain microvessels but not in the peripheral microvessels, the location of which is appropriate for the entry of meningitis-causing *E. coli* into the brain; 2) Caspr1, with an long extracellular domain, is localized at the luminal side of the brain microvessels, which is readily to be exploited by the circulating bacteria when bacteremia occurred at the beginning of meningitis; 3) The N-terminal laminin-G domain of Caspr1 specifically interacted with bacterial virulence factor IbeA, which enables the subversion of host cell signaling by *E. coli* for bacterial internalization. As a result, the released IbeA derived from circulating *E. coli* is able to hijack host Caspr1 to alter the intracellular signaling to enable bacterial penetration from the peripheral blood into the brain.

The survivors of bacterial meningitis often sustain neurologic sequalae, likely caused by the neuronal apoptosis in the brain. In the past decades of studies, the apoptosis of neurons during bacterial meningitis was naturally considered to be the secondary events caused by the inflammatory responses accompanied with meningitis. Our results showed that *E. coli* was able to invade into hippocampal neurons leading to their apoptosis, indicating an alternative pathway for apoptosis of neurons during bacterial meningitis. The findings that IbeA-Caspr1 interaction is necessary for* E. coli* invasion into hippocampal neurons raised an interesting idea that *E. coli* utilize the same receptor, Caspr1, for its penetration through the BBB and then invasion into neurons in meningitis. An unexpected “two-birds-with-one-stone” mechanism was revealed that meningitis-causing* E. coli* exploit one unique receptor to invade distinct host mammalian cells.

